# Microcirculatory changes during hyperoxia in a porcine model of ruptured abdominal aneurysm

**DOI:** 10.1186/cc9503

**Published:** 2011-03-11

**Authors:** I Cundrle, V Sramek, P Suk, J Hruda, J Krbusik, M Helan, M Vlasin, M Matejovic, M Pavlik

**Affiliations:** 1 St Anns University Hospital Brno, Czech Republic; 2 University of Veterinary and Pharmaceutical Sciences, Brno, Czech Republic; 3 University Hospital Plzen, Czech Republic

## Introduction

Our goal was to evaluate the effect of hyperoxia on sublingual and ileostomal microcirculation during hemorrhagic and reperfusion shock in a porcine model simulating the rupture of an abdominal aortic aneurysm (AAA). We wanted to test the effect of hyperoxia on these two vascular beds because hyperoxia is known to cause different arteriolar responses [1].

## Methods

Pigs were randomized into four groups: HEM *n *= 11, HEM-HYPEROX *n *= 11, SHAM *n *= 6, SHAM-HYPEROX *n *= 5. Hyperoxia (FiO_2 _1.0) started 1 hour after hemorrhagic shock and was maintained until the end of the experiment. Microcirculation was recorded with SDF imaging (MicroScan Video Microscope) in eight time points during the whole experiment (T0 before bleeding, T1 to T4 every hour of the 4 hours bleeding, T5 2 hours after the volume was reinfusioned and aorta clamped, T6 after 2 hours of declamping, and T7 after 11 hours of intensive care). In every time point, recordings were sampled three times at 20-second intervals sublingually and from ileostoma. Videodocumentation was elaborated with software AVA 3.0 by single blinded investigator. The following vessel density parameters (TVD, PVD, De Backer score), perfusion parameters (PPV, MFI) and heterogeneity index for MFI and TVD were monitored. Nonparametric statistic methods were used for the evaluation (Statistica 9 CZ). The Mann-Whitney U test was used for comparison of sublingual and ileostomal microcirculations.

## Results

Sublingually there was a significant increase in density parameters TVD and PVD and a decline in TVD heterogeneity index in T5 (end clamping) in the hyperoxia group (*P *< 0.05). In ileostoma there was a significant decline in density parameters TVD in T3 (3 hours bleeding) and De Backer score in T3 and T4 (end bleeding) and in perfusion parameter MFI in T4 in the hyperoxia group (*P *< 0.05). The rest of the parameters remained unchanged. There were no statistically significant changes when comparing sham and sham-hyperoxia groups both sublingually and in ileostoma.

## Conclusions

In this model of ruptured AAA it seems that hyperoxia might compromise microcirculation during bleeding and improved it during resuscitation.
